# Supplementary material for: Emotional Competence Mediates the Relationship between Communication Problems and Reactive Externalizing Problems in Children with and without Developmental Language Disorder: A Longitudinal Study
Source: Int J Environ Res Public Health. 2020 Aug 18;17(16):6008. doi: 10.3390/ijerph17166008 (PMC7459595; doi:10.3390/ijerph17166008)
Supplement: Supplementary file 1 [file ijerph-17-06008-s001.pdf]

Supplement Table S1. Goodness of fit (AIC and log likelihood test) of the different models.

Model	Reactive aggression	Proactive aggression	ODD symptoms	Emotion recognition	Anger dysregulation
**Basic means model**	405.5	-566.4	450.9	1037.6	1285.5
With addition of age and control variables:					
**1. Age**	396.9**	-579.3***	439.5***	1036.8	1261.3***
**2. Gender**	394.8*	-580.4	440.5	1038.7	1261.8
**3. Socio-economic status**	396.8	-577.3	441.5	1036.3	12.61.3
Controlling for Performance IQ (PIQ):					
**Best model without children with missing PIQ:**	Model 2: 375.6	Model 1: -564.3	Model 1: 424.2	Model 1: 978.7	Model 1: 1217.5
**4. PIQ**	376.3	-569.1**	424.9	979.0	1216.5
**4a. PIQ and Gender**	-	-571.1*	-	-	-
**5. Diagnosis * PIQ**	379.7	-576.1*	425.1	978.0	1217.6
With control variables and diagnosis:					
**6. Diagnosis**	395.4	-572.7	436.8*	1015.1***	1260.6
**7. Diagnosis*age**	395.7	-572.4	437.0	1015.8	1261.8
With control variables and Emotional competence (EC), compared to:	Model 2	Model 4a	Model 6		
**8. Emotion recognition**	393.8	-569.5	403.0***		
**9. Diagnosis x emotion recognition**	395.0	-566.6	401.4		
**10. Anger dysregulation**	375.3***	-573.8*	162.8***		
**11. Diagnosis x anger dysregulation**	367.8**	-574.8	158.9*		
With control variables and emotion communication problems (CAM):					
**Best model, without children with missing CAM data:**	Model 2: 346.2	Model 4a: -564.2	Model 6: 379.8	Model 6: 923.0	Model 1: 1149.4
**12. CAM**	340.1**	-574.2***	353.7***	871.2***	1122.5***
**13. Diagnosis x CAM**	342.2	-573.4	354.0	872.8	1124.0
With control variables and CCC: general communication score (GCS), or pragmatics:					
**Best model, without children with missing CCC data:**	Model 2: 355.8	Model 4a: -522.0	Model 6: 371.9	Model 6: 874.4	Model 1: 1118.0
**14. GCS**	354.6	-523.5	369.0*	847.9***	1111.9**
**15. Diagnosis x GCS**	348.5**	-526.2*	369.4	848.8	1115.7
**16. Pragmatics**	354.3	-522.6	358.5***	831.8***	1106.5***
**17. Diagnosis x pragmatics**	350.8*	-525.9*	359.4	832.1	1109.0
With control variables, EC and CAM:					

**Best model with EC, without children with missing CAM:**	Model 10: 325.4	-	Model 11: 135.8	-	-
**19. CAM**	325.3	-	135.7	-	-
**20. Diagnosis x CAM**	326.9	-	135.2	-	-
With control variables, EC and CCC:					
**Best model with EC, without children with missing CCC:**	Model 10 DLD only: 202.7	-	Model 11: 123.08	-	-
**21. Semantic problems**	197.7**	-	-	-	-
**23. Pragmatic problems**	200.9	-	122.5	-	-
**24. Diagnosis x pragmatics**	-	-	123.8	-	-

Note. **p* < .05, ***p* < .01, ****p* < .001; For some models a better model fit was found, whereas the added predictor was not significant after bootstrapping. This was the case for: reactive aggression: positive relation with pragmatic problems in children with DLD, but not in children without DLD (model 15), and positive contribution of CAM in addition to GCS*diagnosis (model 16); proactive aggression: negative relation with PIQ in children without DLD, but not in children with DLD (model 5) and positive relation with mean anger dysregulation in both groups (model 10); ODD symptoms: a positive relation with GCS in both groups (model 12).

Supplement Table S2 Pearson's correlations between all variables with in the upper right corner the correlations for all children together and in the bottom left corner the correlations separately for the two groups (children with Developmental Language Disorder (DLD)/ children without DLD).

	**1**	**2**	**3**	**4**	**5**	**6**	**7**	**8**	**9**	**10**	**11**
**1. Reactive aggression**	1	.56***	.33***	-.17**	.27***	-.11	-.04	.03	.10	.10	.19**
**2. Proactive aggression**	**.43***/.65*****	1	.25***	-.07	.12	-.16*	-.13*	-.01	.16*	.14*	.22**
**3. Oppositional Deviance Disorder symptoms**	**.01/.54*****	01/.32**	1	-.30***	-.27***	-.17**	-.05	-.01	.21**	.28***	.36***
**4. Emotion recognition**	-.14/-.17	-.05/-.03	-26***/-.38***	1	-.34***	.13*	.19**	.10	-.42***	-.47***	-.52***
**5. Anger dysregulation**	**13/.40*****	-.01/.15	**67***/.79*****	-.28***/-.40***	1	-.16*	-.07	.09	.17*	.23**	.32***
**6. Age**	-.03/-15*	-.08/-.19	-.12/-.16	.06/.18	-.11/-.17	1		.03	.01	-.01	-.03
**7. Performance IQ**	-.00/-.02	**-.20*/.09**	.10/-.11	-.01/.21*	.04/-.19	**-.27**/ .05**	1	.11	-.41***	-.36***	-.24**
**8. Socio-economic status**	.02/.06	.07/.06	.01/-.02	-.03/.20*	.08/.09	-.27**/-.05	.05/-.09	1	-.27***	-.28***	-.26***

**9. General communication problems**	**-.07/.30****	-.05/. 14	.09/.25*	-.32***/ -41***	.13/.14	.09/-.13	**-.20*/.12**	.00/-.05	1	.96***	.52***
**10. Pragmatic problems**	**-.03/.26***	-.06/.12	.23**/.31**	-.39***/-.50***	.23**/.21*	-.00/-.07	-.09/.04	.04/-.19	.91***/.88***	1	.56***
**11. Emotion communication problems**	.09/.22*	.06/.12	.25**/.43***	-.37***/-.55***	.36***/.35**	-.02/.03	.06/-.13	-.08/-.25*	.14/.32**	.20*/ .41***	1

*p<.05, **p < .01; ***p < .001; Relations in children with and without DLD were significantly different (bold) for reactive aggression with proactive aggression: Z = -2.41*, ODD: Z = -4.55***; anger dysregulation: Z = -2.24*; general communication problems z = -2.67**; and pragmatic problems: Z = -2.08*. Additionally, differences were found for the relations between proactive aggression and ODD: Z = -2.46*; ODD and anger dysregulation: Z = -2.00*; PIQ and age: Z * - 2.46*; PIQ and proactive aggression: Z = 2.20*; and PIQ and general communication problems: z = -2.28*.
